# Effect of Acoustic Pressure on Temozolomide-Loaded Oleic Acid-Based Liposomes and Its Safety to Brain Tissue

**DOI:** 10.3390/ph18060910

**Published:** 2025-06-18

**Authors:** Vasilisa D. Dalinina, Vera S. Shashkovskaya, Iman M. Khaskhanova, Daria Yu. Travnikova, Nelly S. Chmelyuk, Dmitry A. Korzhenevskiy, Vsevolod V. Belousov, Tatiana O. Abakumova

**Affiliations:** 1 Department of Synthetic Neurotechnologies, Pirogov Russian National Medical University, 117997 Moscow, Russia; 2 Neurotechnology Laboratory, Federal Center of Brain Research and Neurotechnologies, Federal Medical Biological Agency, 119435 Moscow, Russia

**Keywords:** liposomes, oleic acid, temozolomide, focused ultrasound, blood–brain barrier, glioma, drug delivery, controlled release

## Abstract

**Background:** Glioblastoma (GBM) is a highly aggressive primary brain tumor with limited therapeutic options, particularly due to the limited blood–brain barrier (BBB) permeability. Nanoparticle-based drug delivery systems, such as liposomes, can prolong drugs’ circulation time and enhance their accumulation within brain tumors, thereby improving therapeutic outcomes. Controlled drug release further contributes to high local drug concentrations while minimizing systemic toxicity. Oleic acid (OA), a monounsaturated fatty acid, is commonly used to enhance drug loading and increase lipid membrane fluidity. In this study, we developed liposomal formulations with optimized temozolomide (TMZ)’s loading and analyze its response to focused ultrasound (FUS). **Methods**: We synthetized OA-based liposomes with different lipid composition, performed physicochemical characterization (DLS, TEM) and analyzed the TMZ loading efficiency. Different FUS parameters were tested for effective OA-based liposomes destruction. Safety of selected parameters was evaluated in vivo by MRI, histological staining and RT-PCR of pro-inflammatory cytokines. **Results**: All the formulations exhibited comparable hydrodynamic diameters; however, OA-containing liposomes demonstrated a significantly higher TMZ encapsulation efficiency and enhanced cytotoxicity in U87 glioma cells. Moreover, it was shown that OA-liposomes were disrupted at lower acoustic pressures (5 MPa), while conventional liposomes required higher thresholds (>8 MPa). A safety analysis of FUS parameters indicated that pressures exceeding 11 MPa induced brain edema, necrotic lesions and elevated cytokine levels within 72 h post-treatment. **Conclusions**: These results suggest that OA-based liposomes possess favorable characteristics, with an increased sonosensitivity for the site-specific delivery of TMZ, offering a promising strategy for glioma treatment.

## 1. Introduction

Low-grade gliomas are the most common subtype of central nervous system (CNS) tumors, with a high rate of relapse and mortality (a 5-year survival rate of less than 30%) [1,2]. Current chemotherapy approaches are insufficient, mostly due to the limited sensitivity, low stability, rapid clearance, poor solubility and high systemic toxicity of the drugs, as well as the limited permeability of the blood–brain barrier (BBB) in the tumor, leading to insufficient drug concentrations. Several approaches have been developed to increase the drug concentration in the tumor in order to reduce systemic toxicity to the body; one of them is nanoparticle-based delivery systems with triggered-systems drug delivery. A controlled drug release can be carried out under the influence of both internal and external stimuli such as pH; the presence of active forms of oxygen; enzymes, for example phospholipases, proteinase or other specific enzymes; temperature; magnetic field; radioactive radiation; light radiation; and ultrasound radiation [3,4,5]. The main limiting factor of internal stimuli is the heterogeneity of tumor cells and tissues. The disadvantage of radioactive exposure is low safety, with a fairly good depth of radiation penetration, while light radiation is the safest, but its use is limited due to the small depth of penetration. Magnetic field and ultrasound radiation have the most safety and effectiveness. All of these approaches have shown their efficacy in the treatment of various tumors (prostate cancer, breast cancer, hepatocellular carcinoma and others), with more than 300 ongoing clinical studies with nanoparticles for therapy against tumors and other diseases [6].

The use of nanoparticles in glioblastoma treatment is a complex task that is primarily limited by the low BBB permeability. Despite the fact that brain tumors are associated with a compromised BBB integrity (abnormal vascular growth, destruction of tight junctions between cells), the permeability through vascular vessels is uneven. A high heterogeneity is associated with the features of tumor growth, intracellular pressure, hypoxia area and other factors [7]. Moreover, glioblastoma is characterized by the diffuse growth of cancer cells into healthy tissue, and the BBB in the peritumoral zone remains mostly intact. These areas cannot be completely surgically removed and should be reduced by the following radio- and chemotherapy.

Temozolomide (TMZ) is the standard of chemotherapy for the treatment of glioblastomas. Its advantage is the ability to penetrate the BBB (20–40% of the concentration in blood plasma), while its main disadvantage is its safety profile [8,9]. TMZ is stable at acidic pH values and is hydrolyzed under physiological conditions (pH 7.4) to the active component 3-methyl-(triazene-1)-imidazole-4 carboximide (MTIC), which further methylates DNA and leads to apoptosis of the tumor cell. MTIC can then be degraded to 5-aminoimidazole-4-carboxamide (AIC) and the methyldiazonium ion that causes DNA damage [10]. To prevent the fast degradation of TMZ and maintain its therapeutic concentration in tumors, nanoparticles could be utilized [11].

Liposomes are an effective drug delivery system, both for hydrophilic and hydrophobic drugs, which have been successfully translated to the clinic [12]. Doxorubicin-loaded liposomes (Caelyx) have demonstrated their efficacy in Phase II trial in combination with TMZ [13]. Various studies have demonstrated that TMZ can be sufficiently encapsulated into liposomes with an average efficiency of 35-45% and have proved its efficacy in comparison with free TMZ [14]. Tejashree Waghule et. al. showed the TMZ-loaded lipid-based nanocarriers significantly prolonged the blood circulation time of TMZ [15]. Nerea Iturrioz-Rodríguez1 et al. discussed different strategies to overcome the limitations of TMZ-loaded nanoparticles, including loading efficiency [16]. Previously, it was shown that various parameters could affect drugs’ loading in liposomes, including specific phospholipid features (acyl chain length, side chains, charge and the phase transition temperature) [17]. Oleic acid (OA), as well as other unsaturated fatty acids (UFAs), was extensively studied as a surfactant or co-solvent for hydrophobic drugs such as TMZ as well as a component of liposomes [18,19]. Previously, it was shown that liposomes with OA demonstrated a better encapsulation efficiency (EE) (more than 60%) [20]. Also, it was shown that oleic acid could enhance the therapeutic effect by suppressing glutathione peroxidase 4 (GPX4) [21]. The important part is that the incorporation of UFA can impact the fluidity and stiffness of lipid membranes and destabilize them, which could be utilized in controlled drug release [22]. A number of studies have previously investigated the effects of OA and other UFAs using physicochemical methods (e.g., X-ray diffraction, calorimetry) as well as molecular dynamics simulations. For example, Katarzyna and Pawel Hac-Wydro showed that UFAs, when present in small amounts, make membranes thermodynamically more stable, while higher concentrations reduce membrane stability [23]. Similarly, Rebecca Notman et al. investigated the effect of OA on the phase behavior of phospholipid membranes and found that OA promotes the fluidization of dipalmitoylphosphatidylcholine (DPPC) bilayers by decreasing the gel-to-liquid crystalline phase transition temperature from 41 °C to 37 °C at concentrations up to ~33 mol% OA [24]. It was also shown that OA has not only a minor condensing effect on the liposomal bilayer but also increases its fluidity and flexibility. Furthermore, Cerezo et al. demonstrated that UFAs lower the temperature at which the transition from lamellar gel and lamellar fluid phases to nonlamellar hexagonal phases occurs in various membranes and induce structural modifications in the bilayers [25].

Focused ultrasound (FUS) is a safe and easily adaptable external stimulus that could improve the effectiveness of nanomedicine in glioblastoma therapy [26]. It can be used in two modes: high-intensity focused ultrasound (HIFU) and low-intensity focused ultrasound (LIFU). The use of thermosensitive components, such as polymers or lipids, can trigger the release of a substance, while non-thermal methods mostly rely on the inertial cavitation effect, which is a mechanical effect. This approach can reduce the systemic toxicity of a drug by controlling its release only in the pathological area, thus increasing the local delivery of the drug. LIFU is another approach to increase the accumulation of drugs, including ultrasound-inert nanoparticles in the brain. The injection of gas-filled microbubbles, followed by transcranial sonication, has proved its efficacy in delivering anticancer drugs (TMZ, doxorubicin, carboplatin, etc.) to brain tumors. This approach has also shown its effectiveness for the delivery of nanoparticles. Thus, an increase in the accumulation of cisplatin particles and particles with paclitaxel was observed when exposed to ultrasound [27,28].

The development of a combined approach that makes it possible to improve modern methods of glioblastoma therapy using nanotechnology is the most rational step to increase the life expectancy of patients. Here we investigated TMZ-loaded liposomes with an increased entrapment efficiency, stimuli-sensitive release and delivery using different application modes of FUS.

## 2. Results and Discussion

### 2.1. Synthesis and Characterization of Oleic-Based Liposomes

Liposomes are promising drug carriers due to their mechanical strength, flexibility, low toxicity and high biocompatibility [29]. Previous studies have demonstrated that liposomal TMZ can significantly improve the accumulation of TMZ in glioma tissue [30]. In this study, we synthesized liposomes with various lipid compositions to develop either neutral or cationic nanoformulations (Table 1). Specifically, we incorporated Dimethyldioctadecylammonium bromide (DDAB) to investigate the effect of cationic lipids on the delivery of TMZ to glioma cells. Additionally, OA was included in the formulations (Table 2) as the previous research showed its potential as a co-solvent of hydrophobic drugs, which could enhance their entrapment efficiency [31].

**Figure 1:**
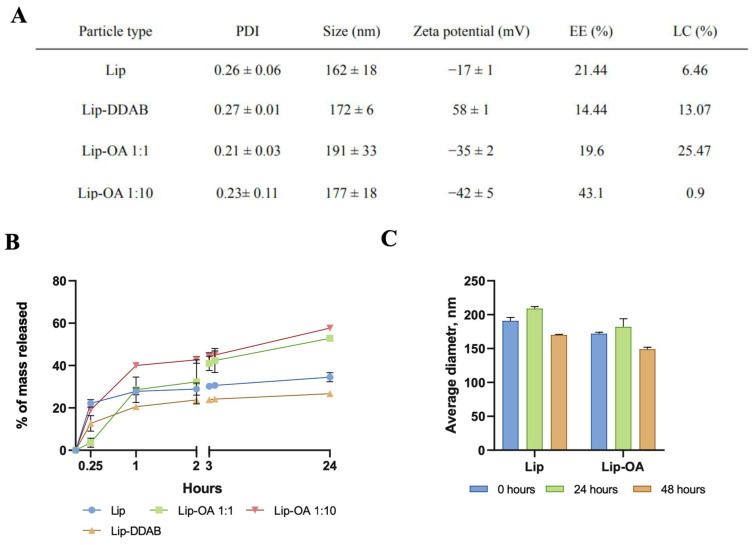
(**A**) Hydrodynamic size, PDI, zeta potential and EE of neutral (Lip), cationic (Lip-DDAB) and OA-based liposomes with different molar ratios to cholesterol (Lip-OA 1:1 and Lip-OA 1:10), mean ± SD, n = 3; (**B**) drug release of neutral (Lip), cationic (Lip-DDAB) and OA-based liposomes in 24 h; (**C**) stability of conventional and OA-based liposome samples measured by DLS in 24 and 48 h, mean ± SD, n = 3, n—number of measurements.

All the liposome formulations exhibited a similar size distribution, with a particle size less than 200 nm. However, the incorporation of OA or DDAB resulted in a slight increase in the hydrodynamic diameter of the liposomes. Notably, a tenfold increase in the molar ratio of OA to cholesterol led to an increase in nanoparticle size compared to the liposomes without OA (from ≈160 to ≈190 nm). Zeta-potential measurements revealed that OA-containing liposomes had a more negative charge compared to the conventional liposomes, while the incorporation of DDAB led to the positive zeta potential of the nanoparticles (Figure 1A). TEM images confirmed that the liposome retained a spherical morphology and had a size of less than 200 nm (Figure S1). Both OA-based and conventional liposomal formulations were stable for up to 48 h (Figure 1C), though OA-based liposomes demonstrated a notable increase in polydispersity index (PDI) after 72 h.

We next evaluated the EE and loading capacity (LC) of the liposomal formulations. To quantify TMZ in different liposome formulations, we compared two methods: mass spectrometry (HPLC-MS) and spectrophotometry. The spectrophotometric method involved measuring TMZ in either the waste solution (collected from the purification step as a non-entrapped drug) or the extracted phase (disrupted Lip-TMZ)—methods commonly used in the literature. Interestingly, spectrophotometric measurements revealed that the calculated EE for TMZ exceeded 60% for all formulations (Table S3), which aligns with other studies. However, HPLC-MS, which provided a more precise measurement of both TMZ and its metabolite (AIC), yielded more reliable results. We found that the EE of TMZ in OA-based liposomes, with a tenfold increase in the OA-to-cholesterol molar ratio, was higher (43.1%) compared to liposomes with equal OA-to-cholesterol ratios (19.6%). For instance, the phospholipid–cholesterol liposomes developed by Jinhua Gao et al. achieved an EE and LC of 35.45 ± 1.48% and 2.81 ± 0.20%, respectively [32]. In this work, the maximums of EE and LC were achieved for OA-based liposomes and were equal to 43.1 ± 2.5% and 0.90 ± 0.03%, respectively.

To further improve the drug loading efficiency of OA-based liposomes, we employed a double-loading approach. Initially, TMZ was incorporated into the lipid mixture as a premix with OA, serving as a co-solvent. The lipid films were then dissolved in the TMZ solution and subjected to sonication for 5 min to encapsulate the drug within the inner aqueous compartment of the liposome. This double-loading method resulted in a twofold increase in EE (39.8%) in OA-based liposomes with an equal molar ratio of OA and cholesterol, compared to the single-loaded liposomes (19.6%). Interestingly, double loading did not significantly affect the EE of TMZ in OA-based liposomes with a tenfold increase in the molar ratio of OA to cholesterol, which remained similar to the single-loaded formulation (43.1% vs. 46.7%), as was detected by HPLC-MS. Notably, double-loaded (DL) liposomes exhibited larger sizes and higher EE and LC values, along with a slower TMZ release, compared to single-loaded (SL) liposomes. Furthermore, DL liposomes with an equal OA-to-cholesterol molar ratio demonstrated a greater cytotoxic effect on U87 glioma cell cultures compared to their single-loaded counterparts (Figure S2C). However, no significant difference in cytotoxicity was observed between the DL and SL liposomes containing a 1:10 OA-to-cholesterol ratio.

### 2.2. In Vitro Cytotoxicity of TMZ-Loaded Liposomes

**Figure 2:**
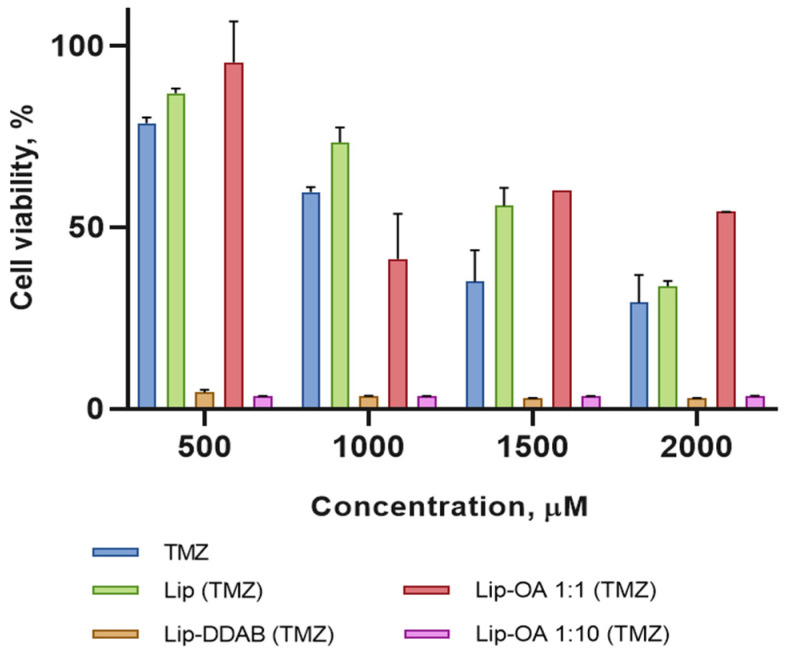
Cytotoxicity of TMZ, conventional liposomes with incorporated TMZ (Lip (TMZ)), OA-based liposomes with incorporated TMZ and equal (Lip-OA 1:1 (TMZ)) and tenfold increase in molar ratio of OA to cholesterol (Lip-OA 1:10 (TMZ)) and cationic liposomes with incorporated TMZ (Lip-DDAB (TMZ)) on U87 glioma cells in 96 h. Four biological replicates per sample were used; results represent mean ± SD.

As the next step in our study, we decided to investigate the cytotoxicity of the obtained nanoformulations. Human U87 glioma cells were treated with the various liposome formulations—neutral (Lip), cationic (Lip-DDAB) and OA-based liposomes with varying OA-to-cholesterol molar ratios—along with free TMZ for a duration of 4 days. The results revealed that the cationic liposomes and the OA-based liposomes with a tenfold increase in the molar ratio of OA to cholesterol exhibited a significantly higher cytotoxicity at all drug concentrations when compared to the control groups. This enhanced toxicity is likely attributable to the increased cellular uptake of these formulations. Interestingly, the conventional liposomes and the OA-based liposomes with an equal molar ratio of OA to cholesterol did not show any significant increase in cytotoxicity when compared to free TMZ (Figure 2). Moreover, both conventional and OA-based liposomes (Lip-OA 1:1), without the encapsulated drug, did not induce toxicity in U87 cells (Figure S3). However, Lip-OA 1:10 in high concentrations slightly decreased cell survival, possibly due to the tumoricidal effect of OA-based liposomes (Figure S3). Jung et al. observed similar effects [33].

Thus, we explored several strategies to improve the loading efficiency of liposomal TMZ, including the incorporation of OA and a two-step loading method. OA has previously been shown to enhance the loading of poorly soluble drugs such as ciprofloxacin, clofazimine and diclofenac [34,35,36]. OA may also enhance the tumor accumulation of TMZ, as supported by studies demonstrating improved drug delivery and survival when TMZ is conjugated with unsaturated fatty acids [37,38,39].

### 2.3. Effect of Acoustic Pressure on Liposome Stability by Focused Ultrasound

FUS can exert multiple effects depending on the acoustic parameters: cavitation, mechanical (non-thermal) effects and thermal effects. For drug delivery applications, cavitation and mechanical effects are most relevant, as they enable a controlled release without damaging surrounding tissues. Several studies have demonstrated that modifying the lipid composition of liposomes can enhance their ultrasound responsiveness. OA is known to increase membrane fluidity by disrupting ordered lipid structures such as raft domains. Onuki et al. demonstrated that incorporating 30% OA significantly enhanced the fluidity of DPPC membranes [40]. In our study, the inclusion of OA in liposomal formulations resulted in a higher LC compared to conventional neutral or cationic TMZ-loaded liposomes, and we supposed that the incorporation of oleic acid could improve its sonosensitivity.

First, we aimed to assess the impact of FUS on the obtained liposome stability; we selected optimal parameters by monitoring the temperature changes in water samples. The temperature increase was kept within a range of 1–3 °C to ensure that the liposomes’ sensitive drug release properties were not compromised (Table S1). Based on these findings, we selected the following parameters for further experimentation: a pulse repetition frequency (PRF) of 5 Hz, pressures of 4.45 MPa and 8.03 MPa, an exposure time of 60 s and a duty cycle of 5%.

**Figure 3:**
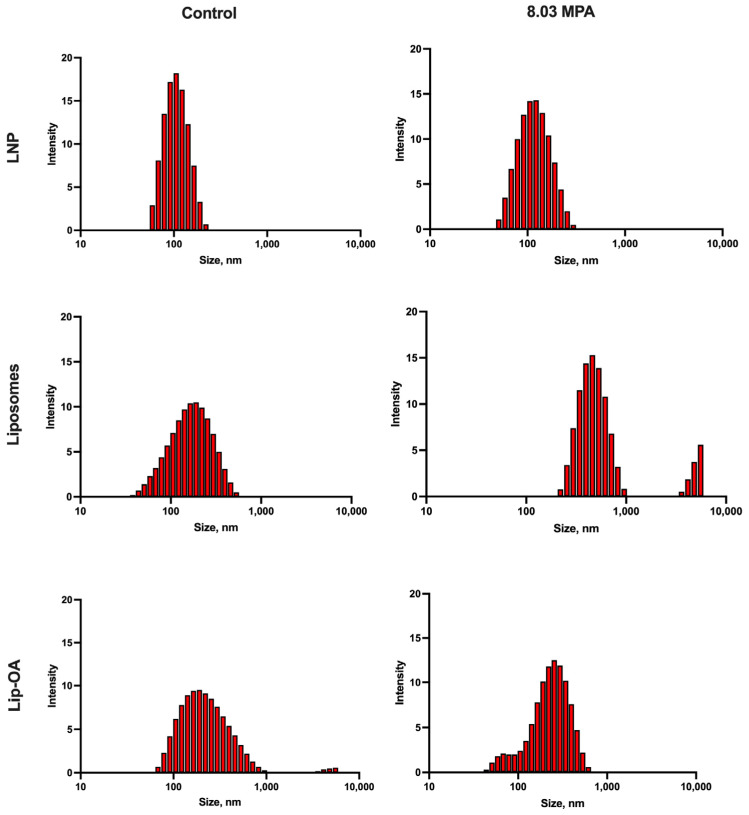
DLS measurements of the size distribution (hydrodynamic diameter) of different types of nanoformulations (liposomes, lip-OA and LNPs) before and after sonication with FUS.

Next, we investigated the effect of these optimized FUS parameters on the structural integrity of liposomes. We compared the behavior of conventional liposomes with OA-based liposomes, and Lipid Nanoparticles (LNPs) were included as a control [41]. The temperature rise during the FUS treatment was monitored (Table S1). Following the 60 s FUS treatment, we assessed liposome destruction using dynamic light scattering (DLS). We observed that at the minimum FUS parameters (1 Hz and 5.83 MPa), no particle destruction occurred in either the LNPs or conventional liposomes, while OA-coated liposomes showed signs of degradation: the value of the maximum size distribution was increased and/or the size distribution was bimodal. At a 5 Hz PRF and 8.03 MPa, both conventional liposomes and OA-based liposomes were significantly disrupted compared to the control group, while LNPs remained intact. At the highest FUS parameters (5 Hz and 11 MPa), all liposomal formulations, including conventional liposomes and OA-coated liposomes, were destroyed, whereas the control LNPs remained stable (Figure 3). These results highlight that the effectiveness of FUS in disrupting liposomes and promoting drug release is influenced by both the PRF and pressure, with OA-based liposomes exhibiting an enhanced sensitivity to FUS compared to conventional formulations.

Thus, it can be stated that the introduction of OA into the composition of lipids for liposomes allows for an increase in their mobility and ability to be destroyed. Similar data were obtained earlier when introducing unsaturated phospholipids into the composition of liposomes. For example, the incorporation of phospholipids with unsaturated acyl chains can destabilize the lipid bilayer, increasing its sensitivity to FUS [42]. The ultrasound-triggered release of cisplatin from modified liposomes has been shown in murine C26 colon adenocarcinoma models. Additionally, the inclusion of PEGylated lipids may further enhance ultrasound sensitivity, possibly by absorbing ultrasonic energy and concentrating it at the liposome surface [43]. Building on this work, our study suggests that OA-containing liposomes may exhibit enhanced ultrasound sensitivity, leading to an improved TMZ delivery to glioma tissue. We observed that OA’s incorporation not only increased TMZ encapsulation but also improved ultrasound-triggered release compared to conventional liposomes.

### 2.4. Safety of Focused Ultrasound on Brain Tissue and Biodistribution of Synthesized Liposomes

In previous studies, we identified optimal FUS parameters that did not induce significant heating of the surrounding tissue (Table S1). These parameters were effective in the destruction of OA-based liposomes in vitro (Figure 3), but their safety profile in vivo remained to be fully assessed. To explore the safety parameters associated with ultrasonic particle destruction, we employed four distinct treatment regimens (Table 3) to investigate the impact of these parameters on brain tissue.

MRI scans were performed at two time points: 3 h and 72 h after sonication. T2-weighted images of the brains of mice (n = 6 per group) were used to assess the presence of any abnormal tissue alterations. At the 3 h, groups 3 (10 MPa) and 4 (11 MPa) showed hyperintense foci on the MRI scans, suggesting the development of edema and inflammation (Figure S5). These findings indicated that tissue damage had occurred shortly after FUS exposure.

**Figure 4:**
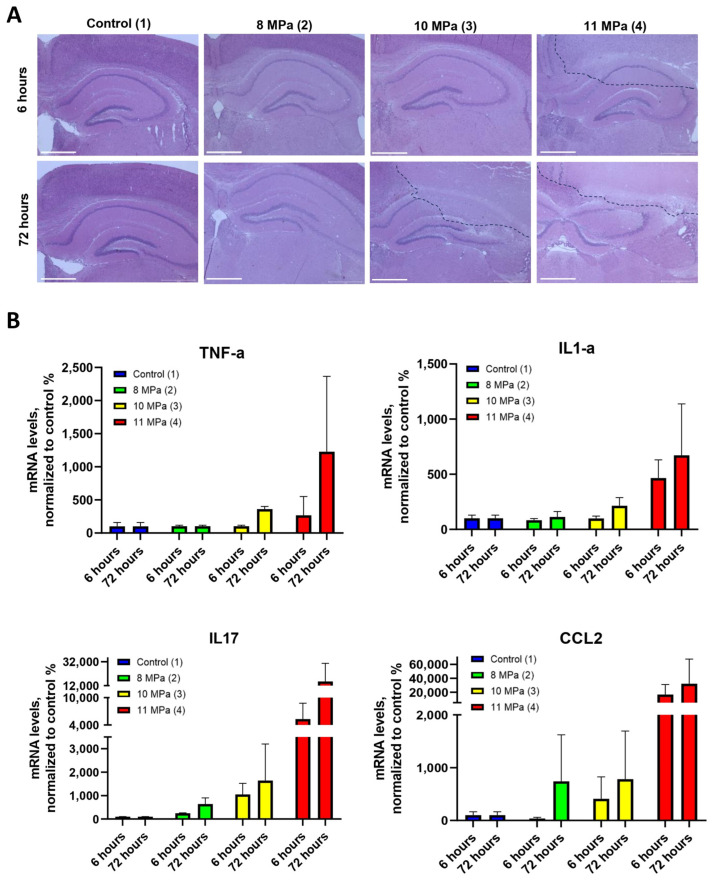
(**A**) Histological images of mouse brain sections after exposure to FUS with various parameters using hematoxylin–eosin staining. Dashed lines indicate the damaged area (including necrosis). Scale bar 650 µm. (**B**) Analysis of mRNA expression of cytokine markers of inflammation, *TNF-α*, *IL-1α*, *IL-17* and *CCL2*, in brain region homogenates after exposure to FUS. Three biological replicates per sample were used; results represent mean ± SD.

For a more detailed pathological assessment, histological analysis was conducted on brain tissue sections stained with hematoxylin–eosin at two time points: 6 h (acute phase) and 3 days (delayed phase) after sonication. At 6 h, higher FUS pressures (10 and 11 MPa) were associated with significant tissue damage, including necrosis, pericellular edema, and localized hemorrhages (Figure 4 and Figure S6). High-magnification images revealed necrosis surrounding the central lesion area, indicating a more extensive tissue injury than what was evident on the MRI scans at this stage. These findings underscore the need for more precise imaging techniques to detect widespread damage at early time points. We also assessed the cytokine expression levels in brain homogenates to measure the inflammatory response to FUS exposure. Specifically, real-time PCR was used to quantify interleukin-1α (*IL-1α*), tumor necrosis factor (*TNF-α*), interleukin 17 (*IL-17*) and chemokine 2 (*CCL2*) cytokine levels. The results revealed a significant increase in the expression of *TNF-α* and *IL-1α* at 72 h post-FUS treatment, particularly at higher power levels (10 and 11 MPa) (Figure 4B). Lower power levels (6 and 8 MPa) induced a less pronounced effect. Additionally, *IL-17* expression peaked at 72 h, with the highest levels observed at 10 and 11 MPa, further suggesting its involvement in the inflammatory response. Similarly, *CCL2* mRNA levels reached their maximum at 72 h, particularly at the highest ultrasound pressures (11 MPa). These cytokine expression patterns corresponded with the MRI and histological findings, confirming the presence of an inflammatory response and immune system activation, particularly in the higher pressure groups (3 and 4). The substantial increase in inflammatory markers indicates the importance of carefully controlling the FUS parameters to avoid excessive tissue damage and immune system activation. This study demonstrated the critical importance of optimizing multiple FUS parameters, including ultrasound power, pressure and exposure time, to balance therapeutic efficacy with tissue safety. These results are crucial for translating FUS-based therapies into clinical practice, ensuring that focused ultrasound can be applied effectively without inducing undue harm to surrounding tissues.

While acoustic pressure can lead to ultrasound-sensitive release, there are different modes with the lowest pressure that could be utilized to deliver developed liposomes. We utilized FUS with Sonovue^®^ microbubbles. We demonstrated that the injection of fluorescently labeled liposomes led to a significant increase in fluorescence in comparison to the perfused brain (Figure S4). The similar results were demonstrated by Zhuqing Song et al., who showed that TMZ-loaded liposomes could be successfully delivered across the BBB using microbubble-assisted FUS [44]. These results confirmed the potential of FUS in glioma therapy to work in two modes as an ultrasound-sensitive release and improved delivery by microbubble-assisted BBB opening.

## 3. Materials and Methods

### 3.1. Materials

Phosphatidylcholine (PC), cholesterol, Dimethyldioctadecylammonium (Bromide Salt) (DDAB) and 1,2-distearoyl-sn-glycero-3-phosphoethanolamine-N-[amino(polyethyleneglycol)-2000] (ammonium salt) (DSPE-PEG 2000) were purchased from Avanti (Avanti Research, Alabaster, AL, USA). Methanol, chloroform, acetonitrile, formic acid, ammonium formate and ethanol were obtained from Chimmed (Moscow, Russia). Dimethyl sulfoxide (DMSO), agarose, phosphate-buffer saline (PBS), penicillin/streptomycin and L-glutamine were purchased from Paneco (Moscow, Russia). 1,1′-Dioctadecyl-3,3,3′,3′-Tetramethylindodicarbocyanine and 4-Chlorobenzenesulfonate Salt (DID) were purchased from Lumiprobe (Moscow, Russia). Temozolomide (TMZ) was purchased from TCI (Shanghai, China). Oleic acid (OA) was obtained from Macklin Reagent Co., Ltd. (Shanghai, China). Sonovue was purchased from Bracco (Milano, Italy). Dulbecco’s Modified Eagle Media (DMEM) was obtained from HiMedia (Mumbai, India). Fetal Bovine Serum (FBS) was obtained from Biowest (Nuaille, France). AlamarBlue^®^ reagent was purchased from Thermo Fisher (Waltham, MA, USA). Hematoxylin and eosin (H&E) and formalin were obtained from Biovitrum (Moscow, Russia). The primers, ExtractRNA, milli-Q H_2_O, M-MLV Reverse Transcriptase kit and PCR Mix-HS SYBR Master Mix containing SYBR Green I dye were purchased from Evrogen (Moscow, Russia). JetSpin™ centrifugal filters were obtained from Biofil (Guangzhou, China). The Acquity UPLC BEH Amide 2.1×*150 mm column was purchased from Waters (Watertown, MA, USA). Dialysis bags (MWCO 3.5 kDa) were obtained from Spectra/Por (SpectrumLaboratories Inc., Rancho Dominguez, California, USA). Zoletil and xylazine were obtained from Virbac (Carros, France). Ultrasound (US) gel was purchased from Geltek (Moscow, Russia). Isoflurane was obtained from Karizoo Laboratorios (Barcelona, Spain).

### 3.2. Cell Culture

GL261 cell lines were kindly provided by Dr. Aleksei Stepanenko from the Department of Fundamental and Applied Neurobiology of the V. P. Serbsky Federal Medical Research Center of Psychiatry and Narcology. The U87 cell line was obtained from ATCC. Cell cultures were grown in DMEM supplemented with 10% FBS, penicillin (500 units/mL), streptomycin (500 mkg/mL) and L-glutamine (0.4 M) in a 37 °C humidified incubator with 5% CO_2_. Additionally, U-87 cells were cultured in collagen-coated plates. Each well in a 96-well plate was filled with 60 µL of Collagen Type I (32 µg/mL) and incubated for 30 min (37 °C, humidified 5% CO_2_ atmosphere). Then, the remaining collagen was removed, and the wells were washed with PBS twice.

### 3.3. Setting up the Calibration Curves of TMZ and AIC

The standard concentration of TMZ (1 mg/mL) was diluted in PBS to stock concentrations from 1 to 100 μg/mL and measured for absorbance at 269 and 329 nm using a fluorescent microplate reader (Varioskan Lux, Thermo Fisher, Waltham, MA, USA). The calibration curves were plotted by Prism 8 (GraphPad software, La Jolla, CA, USA).

### 3.4. Synthesis of Liposomal Formulations

#### 3.4.1. Synthesis of Liposomes (Lip)

Two types of liposomes were synthesized by mixing cholesterol (1 mg), PC, DSPE-PEG 2000 and DDAB at different molar ratios (Table 1).

Combined lipids were dissolved in 5 mL of chloroform/methanol (5:1) solution. The mixture was stirred for 1 h on a magnetic stirrer at 750 rpm, and then the solvent was removed by rotary evaporation. The formed lipid film was hydrated by adding 5 mL milli-Q H_2_O and 5 mg of TMZ dissolved in 150 µL DMSO. Then the mixture was placed in an ultrasound water bath for 10 min at 45 °C. The obtained liposomes were concentrated by ultrafiltration using JetSpin™ centrifugal filters (50 kDa) and stored for 4 °C until further use.

#### 3.4.2. Synthesis of OA-Based Liposomes (Lip-OA)

To evaluate the effect of OA on TMZ loading, different amounts of OA were used. Liposomes were prepared by mixing cholesterol (1 mg), PC, DSPE-PEG 2000, OA and 8 mg TMZ (pre-dissolved in DMSO) at different molar ratios (Table 2).

Lipids were dissolved in chloroform/methanol solution (5:1) followed by evaporation under reduced pressure. The formed lipid films were hydrated by adding 5 mL milli-Q H_2_O, and for core shell liposomes 8 mg of TMZ was added. The films were then sonicated for 10 min. The obtained liposomes were concentrated by ultrafiltration using JetSpin™ centrifugal filters (30 kDa) and stored at 4 °C until further use.

### 3.5. Physicochemical Characterization of Liposomes

The hydrodynamic diameter and polydispersity index (PDI) of the liposomes were measured by dynamic light scattering (DLS) using a Zetasizer Nano-S (Malvern Instruments, Worcestershire, UK). Appropriate dilutions were made in PBS. The zeta potential was determined by assessing the mobility of the particles diluted in milli-Q H_2_O in an applied electric field using a Zetasizer Nano-S (Malvern Instruments, Worcestershire, UK). It was then calculated from the electrophoretic mobility based on the Helmholtz–Smoluchowski equation.

The concentration of TMZ and AIC was calculated according to their absorbance calibration curves by measuring the absorbance at 269 nm and 329 nm as described earlier.

Entrapment efficiency (EE, %) was calculated as the percent ratio of entrapped drug to the mass of drug loaded during synthesis via the following equation:

$$\mathrm{EE}=\frac{m_{loaded}-m_{encapsulated}}{m_{loaded}}\times 100$$

where *m_loaded_* is the mass of temozolomide added during synthesis, and *m_encapsulated_* is the mass of total drug (AIC and TMZ) determined with HPLC-MS.

### 3.6. Transmission Electron Microscopy (TEM)

Experiments were conducted using a JEOL JEM-1400 microscope (JEOL, Tokyo, Japan) operated at a 120 kV acceleration voltage. Overview images were taken in conventional bright-field transmission mode. Samples were prepared by casting and evaporating a droplet of solution onto a carbon-coated copper grid (300 mesh). The samples were contrasted with 2% uranyl acetate solution for negative staining. The average diameter of the liposomes was calculated from TEM images using ImageJ software (https://imagej.net/ij/download.html, accessed 10 September 2022).

### 3.7. High-Performance Liquid Chromatography–Mass Spectrometry (HPLC-MS)

To measure TMZ and AIC we have designed an original HPLC-MS method that utilizes hydrophilic interaction chromatography. An Ultimate 3000 UHPLC system (Thermo Scientific, Waltham, MA, USA) was coupled to the Acquity UPLC BEH Amide 2.1 × 150 mm column (Waters, Watertown, MA, USA) and a Q-Exactive HF mass spectrometer working in positive ionization mode (Thermo Scientific). Chromatographic separation was accomplished in the following gradient of A and B solvents—0–2 min: 1%B, 2–11 min: linear ramp from 1 to 50%B, 11–16 min: 50%B, where A was acetonitrile containing 5% water, 0.1% formic acid and 10 mM ammonium formate, and B was water containing the same concentrations of formic acid and ammonium formate as A. Dilution series of the TMZ and AIC standards were prepared in 80% methanol in the 0.5 to 8.0 µg/mL concentration range with 2× steps and analyzed together with the samples to build calibration curves. Each sample and calibration curve point were 100× diluted with solvent A just before analysis and spiked with internal standard (theophylline) solution in 80% methanol to a final concentration of 120 nM. Mass chromatograms were collected in parallel reaction monitoring mode for the precursor ions with mass numbers 195.1 Th, 127.1 Th, 180.1 Th for TMZ, AIC and IS, respectively, isolated in a 1.6 Th mass window. Preliminary studies have shown that under selected fragmentation conditions, the most intense fragments in the MS2 spectra of the above precursors have mass numbers of 138.041 Th, 110.035 Th and 124.051 Th, respectively. Therefore, they were selected as quantifiers for our analytes. The extracted ion chromatograms for these fragments were built, and their chromatographic peak areas were determined using FreeStyle 1.8.2 software (Thermo Scientific) for all the samples and standard solutions. The quotient of the peak areas of the respective analytes and of the IS was the measure of the analyte quantity in the sample.

### 3.8. Stability Studies and Release Studies

#### 3.8.1. Storage Stability

The colloidal stability of the liposomes was assessed at 4 °C (nanoparticle size and polydispersity) for a time period of 48 h. At each time point, small aliquots were diluted and analyzed by DLS.

#### 3.8.2. In Vitro Release Study

An in vitro release study was performed using dialysis bags (MWCO 3.5 kDa). An amount of 1 mL of liposome suspension was loaded into the bags and dialyzed against 20 mL of 0.01 M PBS buffer (pH 7.4) at 25 °C and 250 rpm. At 0.4, 0.5, 1, 2, 3, 4, 24 h, 300 µL samples of the buffer were taken to measure absorbance intensity at 269 nm and 329 nm as described earlier. TMZ and AIC concentrations were calculated using their absorbance calibration curves. Time–% of released drug curves were plotted using Prism 8 (GraphPad software, La Jolla, CA, USA).

### 3.9. In Vitro Cytotoxicity Assay

The cytotoxicity of TMZ and TMZ-loaded liposomes was studied on U-87 cell cultures using the AlamarBlue^®^ Assay according to the manufacturer’s protocol. Cells (3000 cells per well) were seeded in 96-well plates, and at 24 h they were treated with either free TMZ or liposomes and incubated for 96 h. After that the fluorescence was measured on a 560/590 excitation/emission filter using a fluorescent microplate reader (Varioskan Lux, Thermo Fisher, Waltham, MA, USA). Concentration–response curves were plotted using Prism 8 (GraphPad software, La Jolla, CA, USA).

### 3.10. Selection of Parameters for Liposomes and LNP Destruction by FUS

Agarose-based phantom was prepared to assess the parameters needed for the destruction of LNPs by FUS. For that, 2% agarose in distilled water was prepared in a 6-well plate, and small cavities were made by a 0.6 µL Eppendorf for loading the water and thermocouple to measure the differences in temperature by FUS. The total exposure time and duty cycle were constant and comprised 60 sec and 5%, respectively. The Pulse Repetition Frequency (PRF) was 1 and 5 Hz, and the ultrasonic power varied from 6 to 15 V, which corresponds to a pressure from 4.45 to 10.95 MPa.

### 3.11. Ultrasound Disruption of Liposomes and OA-Based Liposomes

The selected parameters were used to assess the destruction of LNPs by FUS. For that, a 2% agarose-based phantom with small pores for LNPs was prepared. The thermocouple was used to measure the temperature rise in the pores during the LNPs’ destruction. The focused distance and duty cycle were 8 mm and 5%, respectively. The PRF was 1 and 5 Hz at different pressures (5.11, 5.83, 7.30, 8.03, 9.13, 10, 10.59 and 11 MPa), with the exposure time 60 s. The PDI of destroyed LNPs and graphs were constructed using Zetasizer Nano-S (Malvern Instruments, UK).

### 3.12. In Vivo Experiments

#### 3.12.1. In Vivo Safety Experiment of Parameters of FUS

All experiments were approved and conducted in accordance with the institutional guidelines of Pirogov Russian National Research Medical University (Moscow, Russia, Approval 08/2023). Several parameters of FUS were assessed in vivo for further use for delivery liposomes with TMZ by microbubbles without inducing any pathological changes. The Fvb mice were anesthetized (Zoletil (tiletamine + zolazepam) 20 mg/kg, xylazine 0.2 mg/kg), the heads were shaved, and then a small amount of US gel was applied. After that the FUS (several parameters) was applied to the heads of the mice (Table 3). To provide a comprehensive understanding of the influence of FUS on the brain tissue, the cytokine expression was analyzed by RT-PCR. The tissue integrity was assessed by MRI, and the overall biological safety was evaluated through histological analysis.

#### 3.12.2. Magnetic Resonance Imaging (MRI)

Magnetic Resonance Imaging (MRI) was conducted on mice three hours and three days after FUS exposure. The animals were anesthetized with 1.3% isoflurane during the procedure. The MR imaging utilized a T2 Turbo Spin Echo mode, with parameters set at TE/TR of 46/3720 ms, a slice thickness of 0.5 mm and a resolution of 384/288 for the transversal plane. The imaging was carried out on a ClinScan 7T magnetic resonance tomography system (Bruker Biospin, Billerica, MA, USA).

#### 3.12.3. Histology

To collect the brain, the mice were anesthetized (Zoletil (tiletamine + zolazepam) 20 mg/kg, xylazine 0.2 mg/kg), and then intravascular perfusion with 20 mL of PBS and then with 20 mL of 10% neutral-buffered formalin was performed. The collected brain samples were fixed in formalin for 24 h. Following this fixation, the samples were rinsed with 70% ethanol before being embedded in paraffin using an embedding device. From the resulting paraffin blocks, two micrometer thick serial sections were sliced and subsequently stained with H&E dyes. These stained sections were then analyzed under a light microscope, the Evos M5000 (Thermo Fisher Scientific, Waltham, MA, USA).

#### 3.12.4. Tumor Model

Mice (C57/Bl6) were anesthetized (Zoletil (tiletamine + zolazepam) 20 mg/kg, xylazine 0.2 mg/kg), and then glioma GL261 mouse cells (50,000 cells in 5 µL) were injected into the striatum (AP −1.5 mm, ML −1 mm, DV −3 mm) using stereotaxis.

### 3.13. Real-Time Polymerase Chain Reaction (RT-PCR)

Total RNA extraction was conducted using ExtractRNA reagent, in accordance with the manufacturer’s guidelines. A quantity of 1 μg of RNA was reverse-transcribed into cDNA using an MMLV kit with random primers. The temperature for this process was set at 60 °C, and the duration was one hour. All quantitative polymerase chain reaction (qPCR) experiments were performed using the primers specified in Table S2. The qPCR reactions were prepared using the LightCycler 96 (Roche, Basel, Switzerland) and qPCR Mix-HS SYBR Master Mix containing SYBR Green I dye. The following PCR cycle parameters were utilized: 95 °C for 180 s, 40 cycles of denaturation at 95 °C for 30 s, annealing at 62 °C for 30 s, and an extension step at 72 °C for 60 s.

The mRNA expression of the genes of interest was quantified using the 2^−∆∆Ct^ method. Data were normalized to the GAPDH mRNA level. The measurements were carried out in three technical replicates for each gene in three independent experiments.

## 4. Conclusions

Ultrasound-sensitive drug delivery systems offer a non-invasive and controlled method to trigger the release of therapeutic agents at the target site, minimizing systemic toxicity and enhancing local drug efficacy. In conclusion, we developed OA-modified liposomal formulations of TMZ with an enhanced drug loading, sustained release, and increased sensitivity to FUS. OA’s incorporation significantly improved EE and cytotoxicity in glioma cells, while enabling an effective drug release at lower ultrasound pressures. There are several challenges and limitations that should be overcome in further development. First, the optimization of the manufacturing process of the obtained OA-based liposome requires a detailed stability study to choose storage conditions, including shelf life, colloidal stability and the chemical stability of lipid components. Another challenge is achieving an optimal drug concentration in glioma tissue. While the therapeutic effect of TMZ-loaded liposomes in glioma-bearing mice has been previously shown, the delivery challenges of these nanocarriers remain. Here we demonstrated that transient BBB opening with microbubbles led to a successful accumulation of TMZ-loaded liposomes in glioma tissue in 24 h after injection. The repeated administration of stimuli-responsive nanocarriers requires precise targeting, temperature control and visualization to provide maximal safety for healthy tissue for further clinical translation. We demonstrated that the incorporation of OA allows one to decrease the acoustic pressure to safer parameters, and it could be utilized in further developments. Our results demonstrated OA-based liposomes as a promising platform for controlled, site-specific TMZ delivery in glioblastoma therapy.

## Figures and Tables

**Table 1:** Lipid composition of control liposomes (molar ratio).

Name	Cholesterol (%)	PC (%)	DSPE-PEG2000 (%)	DDAB (%)
Lip	33.8	62.2	4.8	-
Lip-DDab	29.7	56	4.4	9.9

**Table 2:** Lipid composition (molar ratio) of OA-based liposomes.

Name	Cholesterol (%)	PC (%)	DSPE-PEG (%)	Oleic Acid (%)
Lip-OA 1:1	18.45	61.83	1.27	18.45
Lip-OA 1:10	6.93	23.24	0.48	69.35

**Table 3:** FUS parameters used for safety study in vivo on the brain tissue.

Group	Description	Duty Cycle	Pulse Repetition Frequency (PRF)	Pressure	Total Exposure Time
1	Control	-	-	-	-
2	Focused Ultrasound	5%	5 Hz	8.03 MPa	60 s
3	Focused Ultrasound	5%	5 Hz	10 MPa	60 s
4	Focused Ultrasound	5%	5 Hz	11 MPa	60 s

## Data Availability

The original contributions presented in this study are included in the article/Supplementary Materials. Further inquiries can be directed to the corresponding authors.

## Supplementary Materials

The following supporting information can be downloaded at: https://www.mdpi.com/article/10.3390/ph18060910/s1, Figure S1: TEM images of liposomes: A—10,000×; B—20,000×; Figure S2: (A) Hydrodynamic size, PDI, zeta potential and EE of Dl OA-based liposomes with equal (Lip-OA 1:1 Dl) and tenfold increase in molar ratio to cholesterol (Lip-OA 1:10 Dl), mean ± SD, n = 3. (B) The drug release from Sl and Dl OA-based liposomes in 24 h. (C) The cytotoxicity of TMZ and Dl OA-based liposomes with different ratio of OA to cholesterol on U87 cells, mean ± SD, n = 6; Figure S3: Cytotoxicity of conventional liposomes (Lip), OA-based liposomes with equal (Lip-OA 1:1) and tenfold increase in molar ratio of OA to cholesterol (Lip-OA 1:10) on U87 glioma cells in 96 h. Four biological replicates per sample were used; results represent mean ± SD; Figure S4: Accumulation of cationic liposomes with TMZ labeled with fluorescent dye DiD in the brain (A) and biodistribution of liposomes in the heart, lungs, liver, spleen and kidney (B) after BBB opening by microbubbles using IVIS Spectrum CT. Lip-TMZ indicates the injection of liposomes with TMZ without induction of FUS. Lip-TMZ_FUS indicates the injection of liposomes with TMZ and FUS. Control group indicates a healthy mouse without the injection and induction of FUS; Figure S5: T2-weighted images of the brain of mice in control and after exposure to ultrasound (after 3 h and 3 days); Figure S6: Histological images of mouse brain sections before (A) and after exposure to FUS with 11 MPa (B) using hematoxylin–eosin staining. Arrows indicate the pericellular edema. Scale bar 150 µm. Table S1: The selection of FUS parameters by the differences in temperature; Table S2: The primer sequences used in RT-PCR; Table S3: The values of EE of TMZ measured by mass spectrometry and spectrophotometry (waste and extract) in conventional (Lip), cationic (Lip-DDab), single-loaded (Lip-OA 1:1) and double-loaded (Lip-OA 1:1 DL) OA-based with equal molar ratio of OA to cholesterol and single-loaded (Lip-OA 1:10) and double-loaded (Lip-OA 1:10 DL) OA-based with tenfold increase in molar ratio of OA to cholesterol liposomes.
