# Supplementary material for: Effect of Acoustic Pressure on Temozolomide-Loaded Oleic Acid-Based Liposomes and Its Safety to Brain Tissue
Source: Pharmaceuticals (Basel). 2025 Jun 18;18(6):910. doi: 10.3390/ph18060910 (PMC12195883; doi:10.3390/ph18060910)
Supplement: Supplementary file 1 [file pharmaceuticals-18-00910-s001.zip › pharmaceuticals-3666665-supplementary.pdf]

Table S1. The selection of FUS parameters by the differences in the temperature

Pulse repetition frequency (PRF), Hz	Duty cycle, %	Power, V	Pressure, MPa	Exposure time, sec	Temperature difference, ^{0}C
1	5	6	4.45	60	+0.12
1	5	7	5.11	60	+0.12
1	5	8	5.84	60	+0.18
1	5	10	7.30	60	+0.3
1	5	15	10.95	60	+1.22
5	5	6	4.45	60	+0.94
5	5	7	5.11	60	+1.5
5	5	8	5.84	60	+2.6
5	5	10	7.30	60	+0.7
5	5	15	10.95	60	+4.6

Table S2. The primers sequences used in RT-PCR

Gene Name	Primer Sequence (F-Forward; R-Reverse)<br>(5 '→ 3')
GAPDH	F-TGCACCACCAACTGCTTAGC<br>R-GGCATGGACTGTGGTCATGAG

TNFa	F-CCACCACGCTCTTCTGTCTA<br>R-TCGAATTTTGAGAAGATGATCTGAG
IL1a	F- AAGTCTCCAGGGCAGAGAGG<br>R- ACTGTAGTCTTCGTTTTCACTGT
IL17f	F- CCTGAGGGAAGAAGCAGCCA<br>R- GGAACTGGAGCGGTTCTGGA
CCl2	F-TGTGCTGACCCCAAGAAGGA<br>R-GCATCACAGTCCGAGTCACAC

Table S3. The values of EE of TMZ measured by mass-spectrometry and spectrophotometry (waste and extract) in conventional (Lip), cationic (Lip-DDab), single-loaded (Lip-OA 1:1) and double-loaded (Lip-OA 1:1 DL) OA-based with equal of molar ratio of OA to cholesterol and single-loaded (Lip-OA 1:10) and double-loaded (Lip-OA 1:10 DL) OA-based with tenfold increase of molar ratio of OA to cholesterol liposomes

	HPLC-MS	Spectrophotometry (Waste)	Spectrophotometry (Extract)
Particle	EE, %	EE, %	EE, %
Lip	21,44	99,08	99,12
Lip-DDab	14,44	76,74	63,01
Lip-OA 1:1	15,7±1,1	84,02	60±8
Lip-OA 1:1 DL	31,8±1,5	97,65	>100
Lip-OA 1:10	34,33±1,67	87,51	>100
Lip-OA 1:10 DL	37,39±2,42	98,26	>100

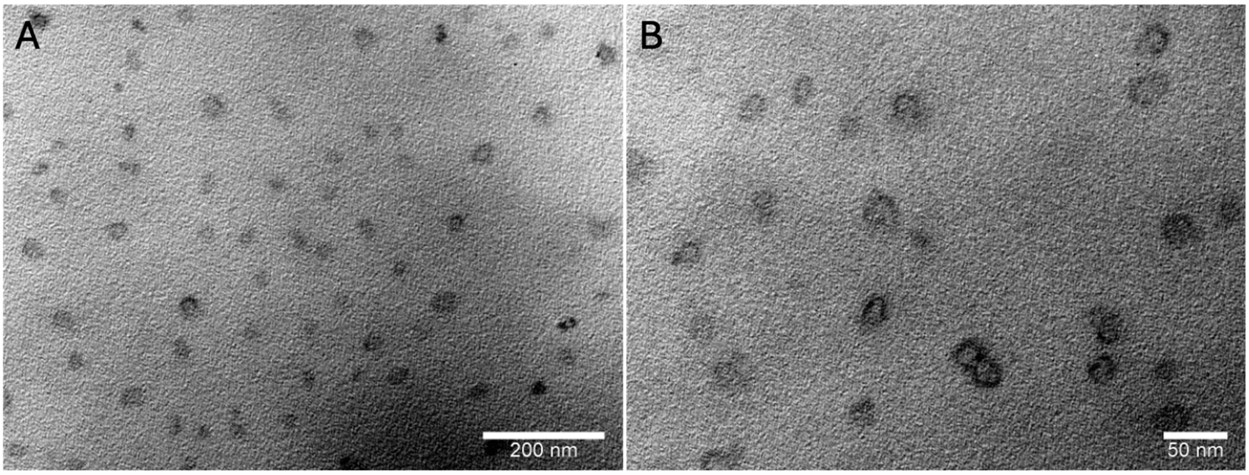

Figure S1. TEM images of Lip: A - 10000x; B - 20000x.

A

Particle type	PDI	Size (nm)	Zeta potential (mV)	EE (%)
Lip-OA 1:1 Dl	0.19 ± 0.01	151 ± 3	-36 ± 1	39.8
Lip-OA 1:10 Dl	0.23 ± 0.11	185 ± 38	-41 ± 1	46.7

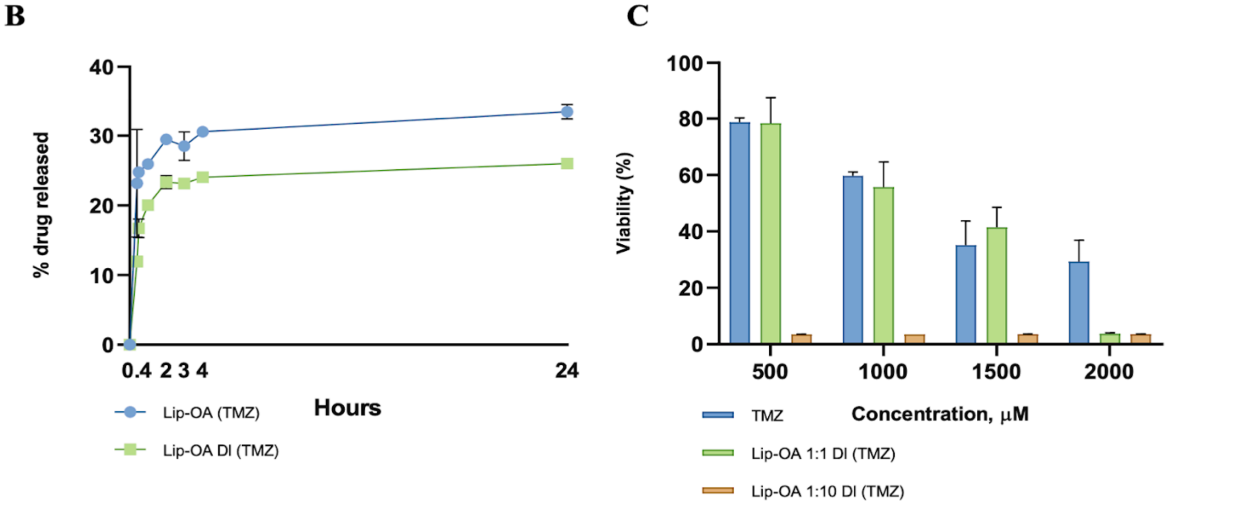

Figure S2. (A) Hydrodynamic size, PDI, zeta-potential and EE of Dl OA-based liposomes with equal (Lip-OA 1:1 Dl) and tenfold increase of molar ratio to cholesterol (Lip-OA 1:10 Dl), mean ± SD, n = 3; (B) The drug release from Sl and Dl OA-based liposomes in 24 hours; (C) The cytotoxicity of TMZ and Dl OA-based liposomes with different ratio of OA to cholesterol on U87 cells, mean ± SD, n = 6.

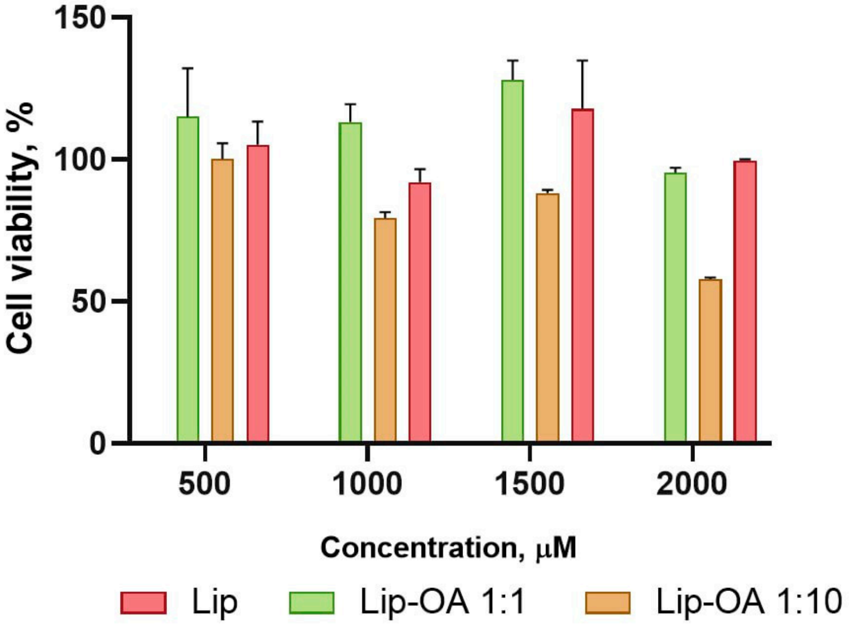

Figure S3. Cytotoxicity of conventional liposomes (Lip), OA-based liposomes with equal (Lip-OA 1:1) and tenfold increase of molar ratio of OA to cholesterol (Lip-OA 1:10) on U87 glioma cells in 96 hours. Four biological replicates per sample were used; results represented mean±SD

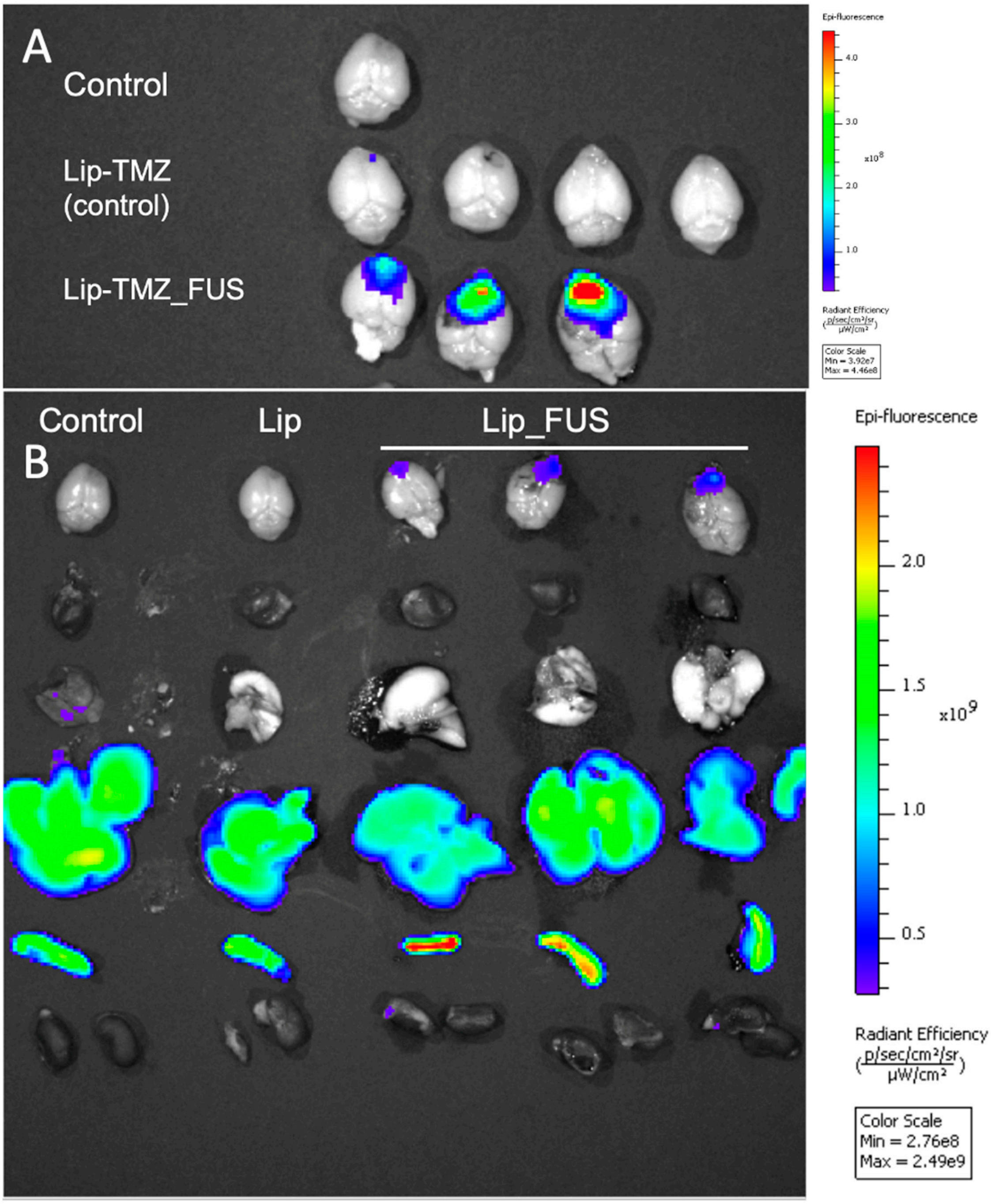

Figure S4. Accumulation of cationic liposomes with TMZ labeled with fluorescent dye DiD in the brain (A) and biodistribution of liposomes in the heart, lungs, liver, spleen and kidney (B) after BBB opening by microbubbles using IVIS Spectrum CT. Lip-TMZ indicates the injection of liposomes with TMZ without induction of FUS, Lip-TMZ_FUS indicates the injection of liposomes with TMZ and FUS. Control group indicates a healthy mouse without injection and induction of FUS.

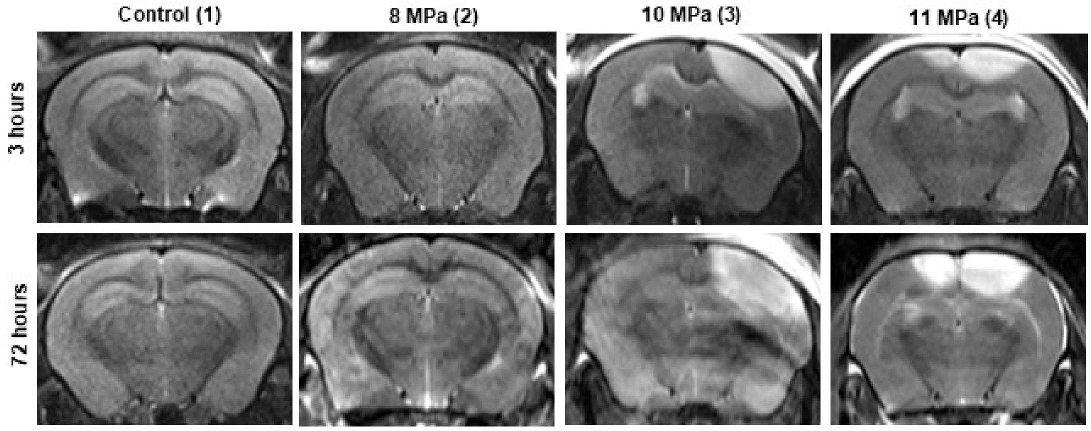

Figure S5. T2- weighted images of the brain of mice in control and after exposure to FUS (after 3 hours and 3 days)

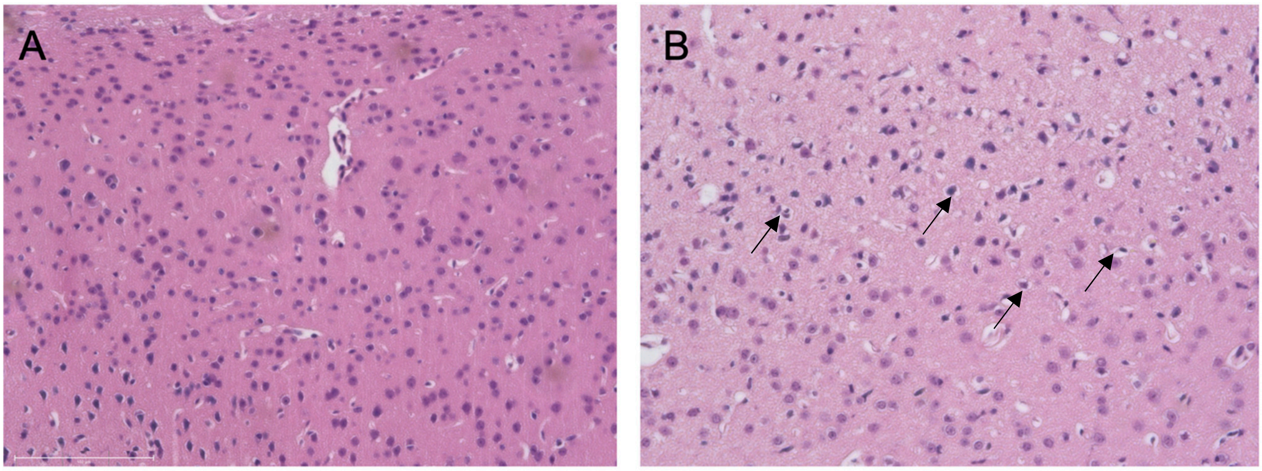

Figure S6. Histological staining (hematoxylin–eosin): control mouse brain (A) and after exposure to FUS with 11 MPa (B). Arrows indicate the pericellular edema. Scale bar 150 µm.
